# A Spotlight on Liquefaction: Evidence from Clinical Settings and Experimental Models in Tuberculosis

**DOI:** 10.1155/2011/868246

**Published:** 2011-03-13

**Authors:** Pere-Joan Cardona

**Affiliations:** ^1^Unitat de Tuberculosi Experimental, Fundació Institut per a la Investigació en Ciències de la Salut Germans Trias i Pujol (IGTP), Universitat Autònoma de Barcelona, Crta de can Ruti s/n 08916, Badalona, Catalonia, Spain; ^2^CIBER Enfermedades Respiratorias, 07110 Bunyola, Mallorca, Illes Balears, Spain

## Abstract

Liquefaction is one of the most intriguing aspects of human tuberculosis. It is a major cause of the transition from the infection to active disease (tuberculosis, TB) as well as the transmission of *M. tuberculosis* to other persons. This paper reviews the natural history of liquefaction in humans from a pathological and radiological point of view and discusses how the experimental models available can be used to address the topic of liquefaction and cavity formation. Different concepts that have been related to liquefaction, from the influence of immune response to mechanical factors, are reviewed. Synchronic necrosis or apoptosis of infected macrophages in a close area, together with an ineffective fibrosis, appears to be clue in this process, in which macrophages, the immune response, and bacillary load interact usually in a particular scenario: the upper lobes of the lung. The summary would be that even if being a stochastic effect, liquefaction would result if the organization of the intragranulomatous necrosis (by means of fibrosis) would be disturbed.

## 1. Liquefaction of Necrotic Tissue Takes Place in the Upper Lobes in Humans

### 1.1. The Primary Infection Is Usually Not Seen

Primary infection is sometimes associated with Ghon's Complex, that is, the presence of a small lesion in the parenchyma, together with enlarged hilar lymph nodes. The primary infection, (about 0.5 mm of diameter in average) is not detected by the radiologist in around 85% of cases [1]. These lesions have been described as nodular-acinar due to their size and location in the bronchial tree, and from a histological point of view, they are granulomas and characterized by the induction of well-encapsulated central necrosis (or caseum) [2]. Such lesions being usually found in necropsies of subjects without any evidence of active TB. They are commonly associated with a “benign evolution” of the infection [3] in which the fibrosis plays a paramount role. Two patterns of fibrosis have been described in these lesions: a central one, based on the production of a collagen matrix to organize a small inert caseum, and a peripheral one, which is the origin of the encapsulation and in which the fibroblasts can be easily identified, and related with the external cellular ring, mostly composed by lymphocytes [3]. Usually, the necrotic tissues of these primary lesions calcify, a fact that has been used in studies aiming to detect infected subjects and/or to evaluate the effectiveness of BCG vaccination in preventing the infection [4]. Interestingly, very few if any calcified lesions carry viable bacilli [5].

### 1.2. The Upper Lobes: the Scenario for Cavitation

Although cavitation in the upper lobes has traditionally been associated with reactivation of old lesions, this image has been found in chest X-ray assay of both recently infected adults or in those with a latent infection (LTBI) that suffered a reactivation [6], thus highlighting the importance of this site in the development of cavitation.

The clinical features observed in immunocompetent adults are the combined result of mycobacterial replication and a destructive host immune response [1]. Thus, the classical findings in chest radiography are upper lobe infiltrates (60%) or cavitary lesions in the lung apex or upper zones of the lower lobes (30–66%) [7], with most patients with pulmonary TB being observed to have multiple cavities [8] with sizes ranging from between 1 cm to more than 5 cm [9]. In contrast, severely immunosuppressed patients, such as HIV positive patients with CD4 < 200 mm^3^, the upper lobe infiltrates and cavitation are reduced to 20% and 10%, respectively [7].

### 1.3. Does the High Oxygen Pressure Favor Liquefaction?

As regards the tropism of cavity formation in the upper lobes, it has been accepted since the 1940s that relative ischemia is likely to affect the apical localization of phthisis in humans. Thus, the very low incidence of progressive apical lesions in patients with mitral stenosis can be explained by the fact that they have a higher pulmonary arterial pressure whereas the very high incidence in patients with pulmonary stenosis is due to the fact that they suffer a global ischemia in the lungs [10]. 

Some years later, West [11] demonstrated that the blood flow in the lowermost pulmonary regions was up to 10 times higher than in the uppermost regions whereas ventilation was only 1.5-times higher, thus generating a progressive fall in the ventilation-perfusion ratio of 3.3 : 0.63 from the apex to the base. This generates large regional variations in the alveolar partial pressures of oxygen, carbon dioxide, and nitrogen, with a difference of 43, 15, and 29 mm of Hg, respectively, and therefore an increase of 41% and a decrease of 39% and 5%, respectively, when compared with the average values. 

On the other hand, the differences as regards blood gas content are much lower because of the shape of the oxygen dissociation curve, the saturation (oxygen content) falling by a 4%. In contrast, the slope of the carbon dioxide dissociation curve varies by 7%, increasing the pH to 7.5, a value outside the normal range for arterial blood. 

All these factors suggest that the bacilli phagocytosed by alveolar macrophages in the upper lobes will have a much higher oxygen tension than those in the lower lobes, thereby favoring their growth [12]. In contrast, the much lower blood flow will reduce both the number of cells to come at the infectious foci and the quantity of bacilli drained by the lymphatic system, thus reducing the immunological surveillance. In addition, once a granuloma is set and local inflammatory response is induced during the course of the infection, the relative alkalosis will reduce the maturation capacity of the dendritic cells, which is stimulated by acidic pH (5.5–7.0), thereby also repressing the development of Th1 immunity [13] (Table 1). 

The impact of a high oxygen pressure in promoting TB has been recently epidemiologically demonstrated by the description of a lower TB incidence in a population living at altitude (3000 m) compared to another one with similar socioeconomic status living at sea level [14]. 

On the other hand, the effect of the ventilation gradient is controversial: the weight of the lung itself can cause some “sagging" within the thorax, the nondependent alveoli being more expanded than the dependent alveoli, much like a “slinky", or coiled spring, hanging under its own weight, where the upper coils are further apart than the lower coils [15]. In contrast, some authors have reported that gravity has only a minor effect on pulmonary blood flow distribution and that two-thirds of the variability in perfusion distribution is determined genetically [16]; this fact could be used as a genetically driven susceptibility to acquire TB.

Moreover, mechanical stress also needs to be considered, as upper lobes have the highest values. Thus, in disease states where the structural integrity of the lung is weakened, mechanical failure occurs at this site. In practice, destruction of parenchyma in centrilobular emphysema is most marked near the apex, with a gradation as we move down the lung. Rupture of a bleb on the upper lobe is almost always the cause of spontaneous pneumothorax in an otherwise healthy young patient [17]. As occurring in the upper lobes, mechanical stress must have a relation with the induction of cavitation (Table 1).

### 1.4. Are Tuberculomas the Consequence of a Failed Liquefaction Process?

A tuberculoma typically appears as a fairly discrete nodule or mass in which repeated infection sites have created a core of caseous necrosis surrounded by a mantle of epithelioid cells and collagen with peripheral round-cell infiltration. The majority of tuberculomas are less than 3 cm in diameter although lesions up to 5 cm have been reported and tend to be mainly found in the upper lobes [18]. Interestingly, both the tuberculomas and the cavitated lesions shown a marked inflammatory response and a high bacillary concentration, but the former having much stronger vascularization and higher proliferative activity, thus indicating a continuous local immune activity [19]. 

Moreover, tuberculomas and cavitated lesions are similar in size [9, 18]. It has been suggested that the former could be an intermediate phase between a controlled or “benign” nodule and a cavitated lesion, with the only difference between them being the liquefaction of the necrotic tissue. Another hypothesis proposes that tuberculomas are actually closed cavities [9].

## 2. The Liquefaction Process Has Only Been Studied in an Experimental Rabbit Model

Tuberculosis in rabbits has often been used as surrogate model for the study of this disease in humans. Unfortunately, the lack of appropriate reagents has led this model to be considered obsolete by immunologists, and logistical problems (i.e., housing and lack of inbred rabbits) have resulted in it being poorly used and underexploited.

The systematic work undertaken by Dannenberg [20], following in the steps of Lurie [21] and working with relatively unsophisticated tools, has provided a great deal of interesting information. The main breakthrough arose as a result of breeding susceptible and resistant rabbit strains (today commercially available rabbits resemble Lurie's resistant strains). Susceptible subjects experienced an earlier increase in bacillary growth and also required a higher bacillary load to trigger an adaptive immune response than the resistant ones, developing in the lesions larger necrosis but (contrary to what would be expected) without liquefaction. This could be due to the fact that they died before these events occurred, or because their macrophages were less activated.

The authors hypothesized on the dichotomy between delayed type hypersensitivity (DTH) and cell-mediated immunity (CMI) to explain these differences in necrosis induction. They considered the susceptible rabbits developed a weak CMI, thus continuing using the tissue-damaging immune response (necrotizing DTH) to stop the intracellular bacillary multiplication. Then, the caseous center enlarges, and local lung tissue is destroyed [20]. Eventually, following infection with virulent bacilli, the susceptible rabbits showed high DTH, because the virulent bacilli reached higher titers in them and therefore provided a larger antigenic stimulus. Nowadays, we know that the DTH response is linked to CMI, being a local manifestation of the arrival of specific lymphocytes attracted by the infected macrophages or, in the case of the tuberculin skin test, those that phagocyte tuberculin [22]. It has been argued that the concentration of the antigens that elicit each is different: Protein Purified Derivative (PPD) elicits DTH in very low concentrations whereas antigens eliciting CMI probably need higher concentrations [20, 23]. On the other hand, the role of specific immunity was clearly linked to the liquefaction process when Yamamura et al. demonstrated that desensitizing with tuberculin-active peptides protected against induction of cavitations in rabbits [24]. This model was also used to demonstrate the more intense the TST the higher the number of cavitations, which was probably due to the larger number of tubercle bacilli in these rabbits [25]. This fact correlates with clinical studies, which showed that patients with larger TSTs were more apt to develop active TB [26]. 

This observation led to the concept that liquefaction required the action of activated or strongly active macrophages, a fact which can nowadays be compared to the situation found in humans suffering from AIDS (see above), who usually do not develop liquefaction and cavitation of the lesions. These findings, therefore, link the action of macrophages to the presence of a specific immune response.

Macrophage hydrolytic enzymes are thought to be responsible for the liquefaction of tuberculous lesions [27]. However, although a number of such enzymes have been identified to date, there is still no direct evidence to confirm that their activity is key to the development of liquefaction as a natural progression of infection.

Finally, experiments showed that keeping *M. tuberculosis*-infected rabbits erect in a harness for eleven hours each day produced cavities in the upper lobes, resembling the situation observed in humans [28].

## 3. The Mouse Model and Its Focus on Necrosis Formation

Typically considered as a resistant host as it is able to survive infection for a long time, nevertheless, the mouse model does not usually develop liquefaction. In fact, we have postulated that these animals are tolerant instead of resistant, as they can harbor a high bacillary concentration without hampering their health status [29]. This can be related with their low capacity to elicit a DTH against PPD, and thus its low induction of intragranulomatous necrosis in this host [30]. Furthermore, the induction of intragranulomatous necrosis in mice has been linked to the genetic background of the animal although this not precluding a better or worse control of the bacillary load, after low-dose aerosol infection. This proposal was based on a comparison between mice with the same H-2 background that develop necrosis but are either more resistant (C57BL/6) or more susceptible (129/Sv) and those that do not develop necrosis but are more resistant (BALB/c) or more susceptible (DBA/2) to the infection [31]. Results showed that 129/Sv mice developed larger necrotic lesions than C57BL/6 and that this was found to be linked to a higher bacillary concentration in the lungs. Using the same infection route, knocking out crucial cytokines or cell types implicated in protection against infection did not prevent the induction of necrosis. Rather, it increased the size of the lesions if the knocking out was related to an increase in the bacillary load [32]. Consolidation of the necrosis was obtained by the chronic phase of the infection (i.e., week 6) in all cases, a fact that could be linked to the accumulation of foamy macrophages (FM), which have been postulated to be the source of the intragranulomatous necrosis [33, 34].

FM have been also related to the drainage of nonreplicating bacilli out from the granuloma through the alveolar spaces, as demonstrated by Sköld and Behar [35], therefore supposed to be a source of constant endogenous reinfection, a factor that has been suggested to be a way of maintaining the Latent Tuberculosis Infection (LTBI) [36]. In this regard, it can be claimed that the mice model is not a good one to reproduce LTBI as it is seen in humans, because it is thought that the main population of latent bacilli resides in the intragranulomatous necrosis, and this phenomenon is weak in mice. This is a controversial issue; as looking at the necrosed tissue in humans [3] or big mammals [37], the presence of bacilli is really very low if present. On the other hand, chronic phase in murine TB has clearly demonstrated the presence of a major population of latent bacilli [38, 39].

Indeed, data obtained in the mouse model and mathematically modulated [40] has shown how, at the onset of infection, bacillary growth occurs in individual alveolar macrophages, and that formation of granulomas is an stochastic process that takes place once a critical number of infected macrophages coincides in a time and spatial manner, and is able to persist after becoming a local spot where enough quantity of chemokines is produced and is stable through time. Then, the granuloma is able to attract specific or innate lymphocytes that would activate the infected macrophages, thus being an important defensive structure against *M. tuberculosis* infection, but that requires a constant chemokine production to persist [41]. These particularities in the onset of the infection provide an important window for constant endogenous reinfection even in the presence of protective immunity [40].

There is one mouse strain (C3HeB/FeJ) able to induce liquefaction of the necrotic tissue, with different outcomes related to the control of the bacillary load [42]. Indeed, if the infection is induced by a low-dose aerosol, this liquefaction can be resumed, and the bacillary load is controlled (Vilaplana et al., in preparation), thus showing an important fact, which is that liquefaction is a reversible process, and thus requires a maintenance whereas it persists, together with a high bacillary load, if the infection is induced by a large dose administered intravenously, causing the death of the animal [42]. Liquefaction has also been observed in SCID mice under certain conditions. Thus, if the animals are treated for a period of time with chemotherapy after infection by aerosol and the infection has been allowed to progress, the bacillary load resumes its growth and leads to liquefaction after suspension of the chemotherapy [43]. This phenomenon can be explained as a result of the sudden and massive resumption of growth in already infected macrophages, which leads to the simultaneous necrosis of them and thus to liquefaction due to an inability to fibrose the necrosis caused. This phenomenon, which we call the “synchronic effect” (Table 1), can help to explain the situation in resistant rabbits and immunocompetent humans, where the sudden onset of the immune response can induce a massive apoptosis and/or necrosis of infected macrophages, thus disturbing the balance of tissue organization and causing its liquefaction. The reason why this does not usually happen in immunocompetent mice could be due to its tolerant response against *M. tuberculosis* infection, thus maybe even when they accumulate a large bacillary concentration, it does not accumulate locally, so the “synchronic effect” does not take place. However, it can explain why some severely immunosuppressed patients develop liquefaction: they are probably suffering from a large necrosis and thus more chances for the synchronic of infected macrophages to take place. 

**Figure 1:**
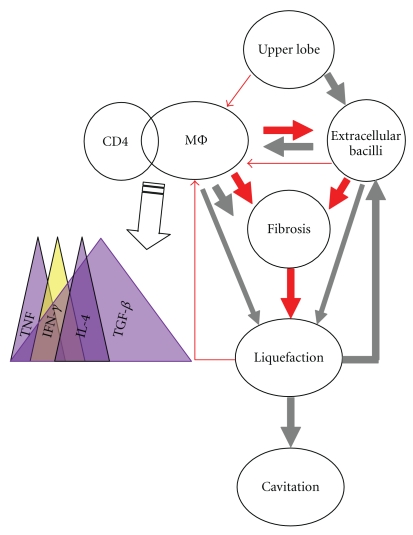
Interactions between the factors involved in the liquefaction process. The colour of the arrows shows the ability to induce a process (in gray) or inhibit it (in red), and the thickness of the arrow is proportional to the intensity of this induction. The upper lobe appears to be the *sine qua non* condition for the process to take place. Macrophage (MΦ) activation and the presence of CD4 is linked to the appearance of different cytokines with time: TNF initially, followed by IFN-*γ* and IL-4, and TGF-*β* from the onset and peaking at the chronic phase. All those cytokines are profibrotic (in violet) except for IFN-*γ* (in yellow). This site mainly undergoes a profibrotic process although there is also a nonspecific antifibrotic effect arising from the macrophages and their enzymatic activity. Extracellular bacilli also have antifibrotic activity and promote macrophage activation although they are also thought to inhibit such activation to some extent. Fibrosis prevents liquefaction whereas liquefaction is promoted by macrophages, the immune response, by promoting the apoptosis of infected macrophages, and extracellular bacilli. Liquefaction induces cavitation, inhibits macrophage activation (indeed, it appears to destroy them), and promotes extracellular bacillary growth. Overall, liquefaction comes first, and then the extracellular multiplication of bacilli occurs. Fibrosis, and thus resume of the liquefaction, would occur only after the number of extracellular bacilli is reduced sufficiently to allow attempts at healing to take place. Finally, a large number of extracellular bacilli results in tissue destruction, cavity formation, and the death of the macrophages that attempt to inhibit such bacillary growth.

Interestingly, the modeling of a Toxoplasma infection in mice, which induces a strong Th1 response, has also provided a further clue by highlighting the delicate balance between TNF and IFN-*γ* in the fibrin deposits in the lesions, with IFN-*γ* clearly favoring fibrinolysis [44, 45], thus again favouring the liquefaction process. This information must be framed in time. It is demonstrated that at the beginning of the *M. tuberculosis* infection, IL-1 and TNF are primary cytokines that favor CMI, and thus the arrival of specific lymphocytes which are able to produce IFN-*γ*, that activates macrophages, so that they can inhibit the growth of tubercle bacilli that they ingest. At the same time, it must be taken into account that TGF-*β* is present from the very onset of the infection, once macrophages phagocyte apoptotic bodies [46], thus downregulating the CMI and favoring the fibrosis, an activity that increases its intensity as the infection progresses, together with the presence of IL-4, which has also been shown to be profibrotic, which also appears at latest time points [47]. This process is shown in the Figure 1 and shows that liquefaction process, only favored by the presence of IFN-*γ* is a process that has a very small window to take place. It is also interesting to note the downregulation induced by TNF to the IFN-*γ* activity which might be a crucial factor to avoid liquefaction [48]. It can be hypothesized that maybe this can be also behind the massive induction of active TB in patients treated with anti-TNF antibodies [49] not only because of the disorganization of the granulomas but for the local increase of IFN-*γ* (without the counterbalance of the TNF) that induces liquefaction.

## 4. Nonhuman Primates

The infection of nonhuman primates has also been shown to produce cavities [23], but in a very different manner when compared to the rabbit model, even if probably reflecting more accurately what happens in humans. Cavitation has been detected as soon as seven weeks after the challenge or later, at 28 weeks after infection although this phenomenon has not been studied exhaustively to date [50], and it can be effectively avoided by BCG vaccination in *Cynomolgus* [51]. Furthermore, it has recently been reported that reactivation of the infection occurs after the decrease in CD4 number induced by the SIV virus, without causing cavitation in the animals, thus resembling what happens in AIDS patients.

## 5. Minipigs Highlights the Role of Fibrosis in Infection Control

Our search for a similarly sized animal to humans with the same degree of intolerance to the bacilli [30] in which an experimental infection could be studied, led to the development of the minipig experimental model. The key point of interest for this model was to reproduce the classical nonvisible lesions of LTBI in humans, qualified as “benign” by Canetti [3]. The results showed two different fibrosis patterns: an inner one, which is well organized due to the influence of myofibroblasts, and an outer one, which was significantly related to the contact with intralobular septae, a structure not present in small mammals (including rabbits and nonhuman primates) and that structure of the lung of big mammals like minipigs (and also humans) [37]. These fibrotic patterns confirmed their similarity with human lesions according to Canetti's descriptions in necropsies. In this model, mature lesions with calcification appeared very rapidly during progress of the infection (week 6 after challenge). Cavitation has also been described in this host [52], thus we do believe the model highlighting the role of fibrosis in the control of the infection, as with no proper fibrosis the liquefaction process occurs readily.

## 6. Do the Bacilli Themselves Interfere in the Fibrotic Process?

It is well known that some components of the cell wall of *M. tuberculosis* are able to bind to plasminogen and even to transform it into plasmin, thereby inhibiting the induction of fibrosis [53]. Likewise, a study in *Streptoccocus pyogenes* showed that the ability to bind to fibrin depends on the specific mammal species concerned, thus it could explain why *M. bovis* is less active against humans than *M. tuberculosis* and vice versa for cows or goats [54]. However, the role of the bacillary bulk is likely to be limited and only relevant when a high extracellular concentration is available in the necrotic and/or the liquefied centre, in this specific way of action, that is, by directly interfering to the fibrin production process. But this process might be paramount for maintaining the liquefaction process and avoiding its reversibility. On the other hand, it has been demonstrated that *M. tuberculosis* specifically upregulates matrix metalloproteinase-1 in human macrophages (whereas BCG vaccine does not) [55]. Excess of MMP activity in the lung, therefore, results in collagen destruction, thus favouring the liquefaction process (Table 1).

## 7. The Role of Architecture

The alveolar spaces in humans and rabbits are much larger than in mice; therefore, this factor could also plays a role in the healing process of the granulomatous lesion. Indeed, it can be argued that the lung parenchyma, being denser in mice, could exert a protective effect by requiring less intense fibrosis to stabilize the granuloma than in humans or rabbits. Effectively, the volume of the alveolar space in humans and rabbits is 57 and 8 times higher, respectively, than in mice [56]. These data clearly show that rabbits and humans must initiate a much faster and stronger fibrotic response than mice to stabilize the necrotic tissue generated inside of the granuloma and also because the necrotic process is much intense. This finding can also be linked to the higher mechanical stress that suffers the upper lobes [17], which interferes in building a fibrotic structure to support the lesion and also may favor liquefaction processes to take place specifically in this site.

## 8. Towards a Holistic View of Liquefaction Induction

Although the role of genetics has been accepted for many years, research in the area of TB induction has mainly focused on the deficiencies induced in the immunological response, especially the Th1-Th2 balance, or nutritional elements that favor mycobacterial growth, such as the availability of iron or the lack of macrophage activation due to reduced levels of vitamin D [57, 58]. Somewhat surprisingly, given the fact that males suffer four times more incidence of active disease than females, the influence of sex was not definitively analyzed until recently [59]. This study suggested that this difference could arise due to the influence of estrogens on the production of IFN-*γ*, or in other words, on immunological factors, as suggested years before [60]. However, the influence of high ferritin concentrations in males, which favors oxidative stress, thereby increasing macrophage activity and possibly inducing fibrinolysis, and thus reducing collagen formation, or the influence of age, which results in increased collagen breakdown and thus a decreased fibrinogenic ability [61], has received little attention.

## 9. What Can Be Done to Further Prevent Liquefaction

Overall, the actual strategies to enhance the specific immune response against *M. tuberculosis* appear to be still good. If considering the dynamic hypothesis of LTBI, a strong cellular immune response must help to control the constant endogenous reinfection of the host. On the other hand, the nature of this immune response (cellular type) leaves a window that makes possible the constant reinfection process [40]. This gives reasons for trying to build a better response to avoid this window by building an effectively protective humoral response, which unfortunately appears to be very difficult although some results have been already obtained in this field [43, 62].

On the other hand, there is the process of induction of tolerance to *M. tuberculosis* antigens, a strategy used for a long time with tuberculins [63] that has proven to be effective in avoiding the liquefaction process, demonstrated by Yamamura et al. experimentally [24]. In this regard, it would be better to have more information about how regulatory T cells behave and how they can be specifically induced to have a more balanced immune response.

## 10. A New Model for Testing Inhibitors of Liquefaction in the Rabbit Skin

Recently, a new experimental model has been validated to test future drugs to inhibit liquefaction [27]. This test is based in the induction of liquefaction in rabbit skin caused by the inoculation of high bacillary load intradermally. The only criticism that can be done is the fact that this phenomenon takes place with another cellular type (i.e., the adypocytes), and it might be difficult to validate its correlation to what it happens in the lungs, but on the other hand, it is a reproducible and reliable test that will sure impulse the knowledge in the field and the research in new tools to avoid the progression towards TB.

## 11. Summary: How Does the Progression to Liquefaction Occur?

Cavity formation has traditionally been considered to occur from solid caseum, and a lot of controversies were raised to understand who is the responsible of inducing liquefaction: the reactivation of the bacilli trapped in the caseum of old lesions or the macrophage through the extracellular release of hydrolytic enzymes.

We understand liquefaction as one of the three possible outcomes (the other two being control and dissemination) of the constant endogenous reinfection process which would maintain LTBI. This idea is supported by human-like experimental models (i.e., the minipig model) suggesting new lesions are constantly generated. The induction of a higher number of new lesions would increase the probability of one of them occurring in the appropriate location to induce liquefaction as upper lobes. These lobes favor higher bacillary load before the immune response appears by directly promoting bacillary growth and delaying the local onset of the immune response. Once this response appears, however, the synchronized induction of apoptosis/necrosis of infected macrophages together with a high IFN-*γ* concentration and the release of metalloproteinases by new incoming macrophages would be critical factors to promote the inhibition of localized fibrosis of the lesion and thus liquefaction. A high ability to generate a nonspecific inflammatory response, which is structurally present in males (i.e., high levels of ferritin), or a lower ability to produce collagen with age, could hypothetically promote this liquefaction.

Although this process can be redirected with time, with fibrosis finally taking place, another, albeit slow factor, namely extracellular bacillary growth, should be taken into account. Such growth might be essential to allow the irreversibility of the liquefaction process already triggered due to the so-called bacillus factor, that is, fibrinolytic properties of proteins from the bacillary cell wall, or by infecting the macrophages that surrounds the liquefaction, and thus maintaining the Th1 response that favors liquefaction to persist, whereby the presence of a large volume of liquefaction product leads to the destruction of new incoming macrophages (due to the high concentration of free fatty acids [20]) and fibroblasts, thereby preventing the structuration of the site. 

As conclusion, it could be said that liquefaction appears to be a stochastic effect due to disturbance in the organization of the intragranulomatous necrosis. The immune response and its magnitude, the bacillary load, the speed of the bacillary growth and the amount of extracellular bacilli, as well as mechanic and chemical factors (due to the distribution of the blood flow) are involved in it. Animal models have provided evidences to infer some of these factors, but more efforts on developing new models should be done in order to better mimic the human disease.

## Figures and Tables

**Table 1:** The factors involved in liquefaction.

(A) The upper lobes are privileged sites
(1) because of their low perfusion/ventilation ratio, which results in:
(a) an increase in bacillary growth inside individual alveolar macrophages due to the high oxygen pressure in the alveolar space,
(b) local alkalosis, and thus inhibition of dendritic cell maturation,
(c) decreased local perfusion, thus delaying the presentation of antigens at the local lymph nodes,
(2) the mechanical stress of ventilation makes stabilization of a fibrotic lesion more difficult.
(B) The fibrinolytic ability of the macrophages.
(C) Immune response
(1) as a result of the synchronic effect, which provokes a massive apoptosis/necrosis of infected macrophages in a short period of time,
(2) induction of high levels of IFN-*γ*, which promotes fibrinolysis,
(3) promotion of a massive entry of macrophages into the lesions.
(D) Extracellular bacillary accumulation
(1) the accumulation of plasminogen and its activation to plasmin induces fibrinolysis and allows the maintenance of the liquefaction
through time,
(2) generation of a mantle of infected macrophages which maintains the inflammatory response.
